# Supplementary material for: Metabolome and Transcriptome Combinatory Profiling Reveals Fluconazole Resistance Mechanisms of Trichosporon asahii and the Role of Farnesol in Fluconazole Tolerance
Source: Microorganisms. 2023 Nov 17;11(11):2798. doi: 10.3390/microorganisms11112798 (PMC10672884; doi:10.3390/microorganisms11112798)
Supplement: Supplementary file 1 [file microorganisms-11-02798-s001.zip › microorganisms-2673574-supplementary.pdf]

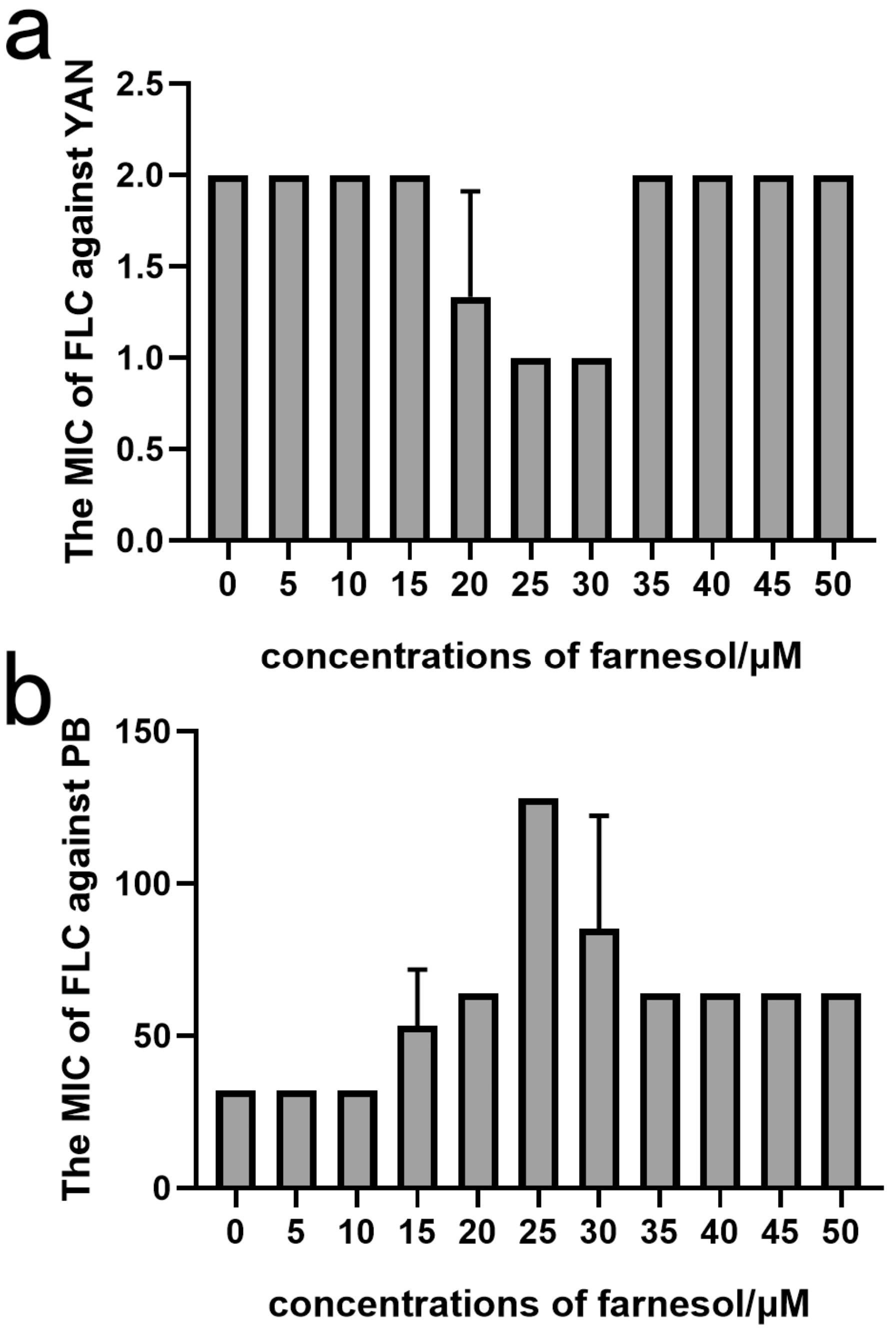

Figure S1. Effect of farnesol concentration on fluconazole resistance.

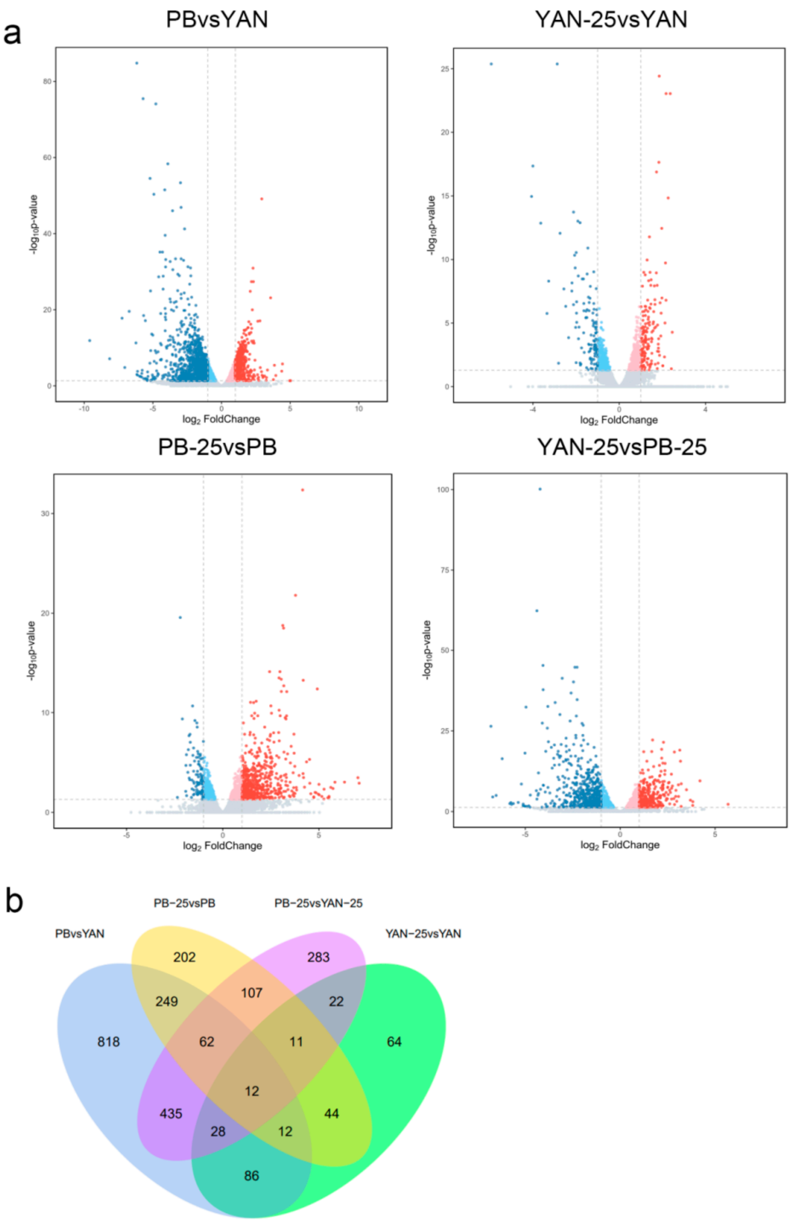

Figure S2. Comparison of DEGs between different pairwise strain combinations. (a) Volcano plots of DEGs among PB vs. YAN, YAN-25 vs. YAN, PB-25 vs. PB, and PB-25 vs. YAN-25. (b) Venn diagram of DEGs among the four pairwise combinations.

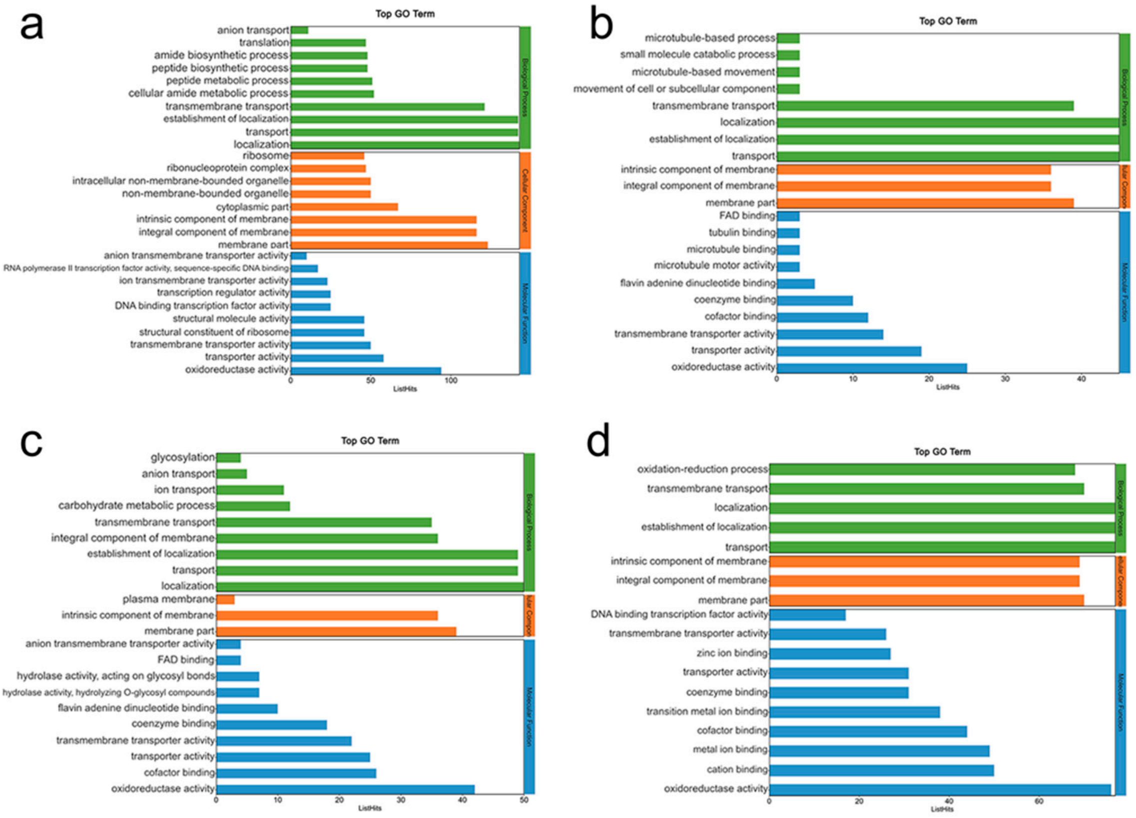

Figure S3. GO classification of DEGs in (a) PB vs. YAN, (b) YAN-25 vs. YAN, (c) PB-25 vs. PB, and (d) PB-25 vs. YAN-25.

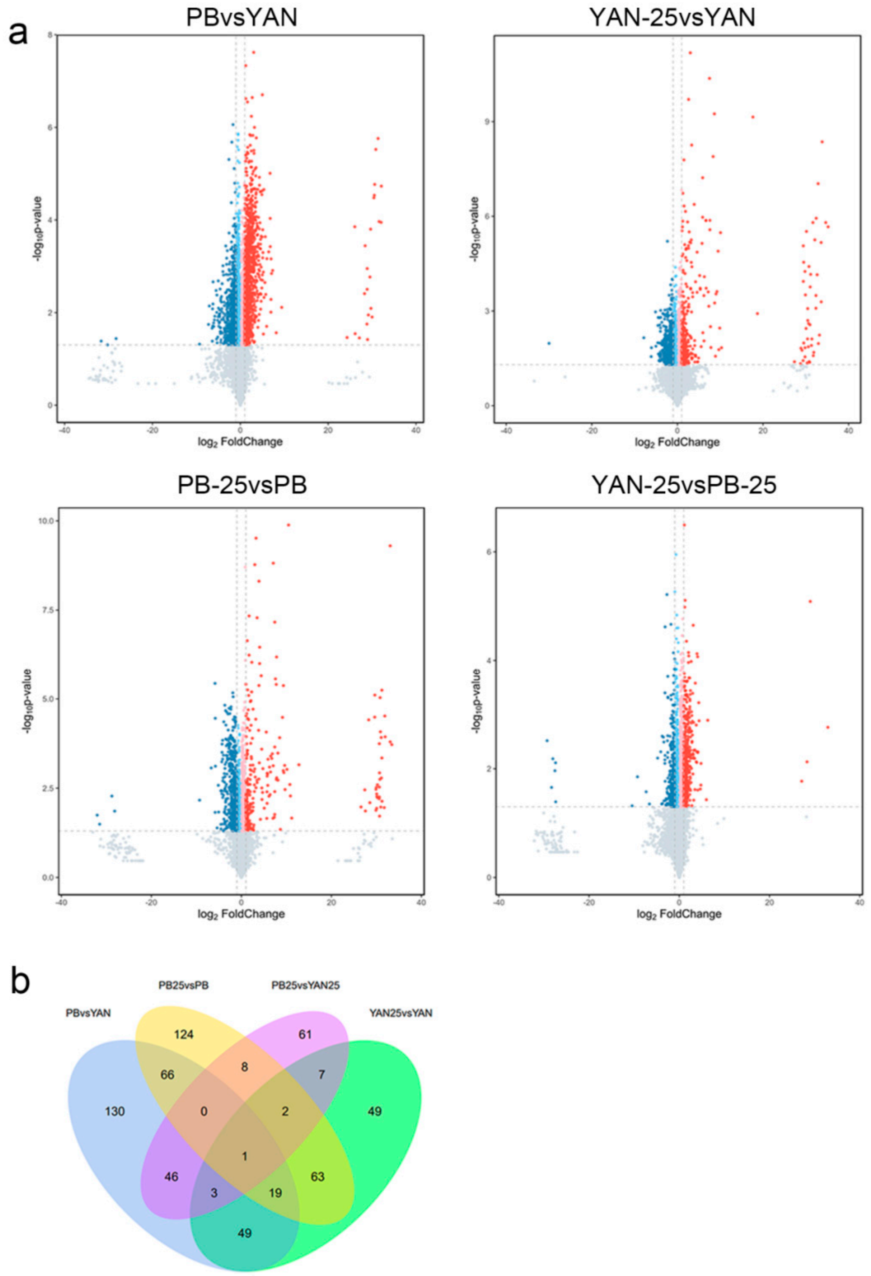

Figure S4. Differentially accumulated metabolites among the three comparisons. (a) Volcano plots of differentially expressed metabolites among PB vs. YAN, YAN-25 vs. YAN, PB-25 vs. PB, and PB-25 vs. YAN-25. (b) Venn diagram of differentially expressed metabolites among PB vs. YAN, YAN-25 vs. YAN, and PB-25 vs. PB.

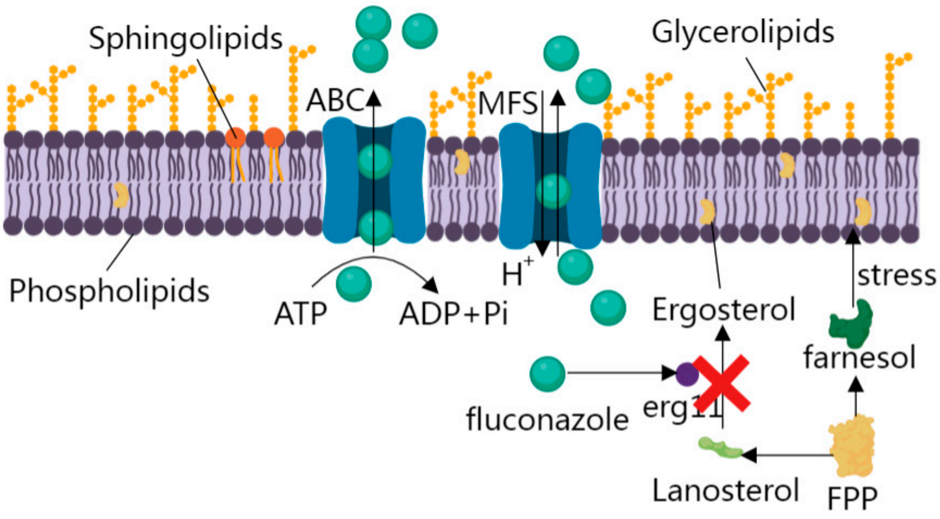

Figure S5. Mechanism underlying membrane lipid and efflux pump regulation that induces fluconazole resistance in fungi.

Table S1. Summary of sequencing reads mapping to the reference genome and quality parameters

sample	library	raw_reads	clean_reads	clean_bases	error_rate	Q20	Q30	GC_pct
YAN_1	FRAS202093395-1r	44871012	42794444	6.42G	0.02	98.19	94.78	62.03
YAN_2	FRAS202093397-1r	44060090	41910974	6.29G	0.02	98.33	95.12	61.65
YAN_3	FRAS202093398-1r	42742116	40836316	6.13G	0.02	98.32	95.06	61.58
PB_1	FRAS202093399-1r	46642798	45090860	6.76G	0.02	98.24	95.01	62
PB_2	FRAS202093400-1r	41592460	40291430	6.04G	0.02	98.18	94.82	62.02
PB_3	FRAS202093402-1r	45470814	43387926	6.51G	0.02	98.24	94.97	62.23
YAN25_1	FRAS202093403-1r	41666358	40312540	6.05G	0.02	98.16	94.64	62.29
YAN25_2	FRAS202093404-1r	52396850	51163252	7.67G	0.02	98.15	94.68	62.24
YAN25_3	FRAS202093405-1r	41860166	39508866	5.93G	0.02	98.23	94.95	62.27
PB25_1	FRAS202093408-1r	47401698	45605730	6.84G	0.02	98.14	94.78	61.88
PB25_2	FRAS202093409-1r	46769642	44507278	6.68G	0.02	98.19	94.91	61.73
PB25_3	FRAS202093410-1r	45790236	44563098	6.68G	0.02	98.12	94.72	61.73

Table S2. Summary of sequencing reads mapping to the reference genome and quality parameters

sample	total_reads	total_map	unique_map	multi_map	read1_map	read2_map	positive_map	negative_map	splice_map	unsplice_map	proper_map
YAN_1	42794444	38702292(90.44%)	38471329(89.9%)	230963(0.54%)	19279369(45.05%)	19191960(44.85%)	19156808(44.76%)	19314521(45.13%)	8434684(19.71%)	30036645(70.19%)	36599776(85.52%)
YAN_2	41910974	37850111(90.31%)	37652252(89.84%)	197859(0.47%)	18852004(44.98%)	18800248(44.86%)	18737267(44.71%)	18914985(45.13%)	7421896(17.71%)	30230356(72.13%)	35779608(85.37%)
YAN_3	40836316	36489795(89.36%)	36285861(88.86%)	203934(0.5%)	18176117(44.51%)	18109744(44.35%)	18035296(44.16%)	18250565(44.69%)	7799035(19.1%)	28486826(69.76%)	34795122(85.21%)
PB_1	45090860	40838034(90.57%)	40580291(90.0%)	257743(0.57%)	20333546(45.09%)	20246745(44.9%)	20246526(44.9%)	20333765(45.1%)	9908331(21.97%)	30671960(68.02%)	38769504(85.98%)
PB_2	40291430	36348389(90.21%)	36114387(89.63%)	234002(0.58%)	18095659(44.91%)	18018728(44.72%)	18020707(44.73%)	18093680(44.91%)	8742555(21.7%)	27371832(67.93%)	34486670(85.59%)
PB_3	43387926	39697615(91.49%)	39472358(90.98%)	225257(0.52%)	19757019(45.54%)	19715339(45.44%)	19693210(45.39%)	19779148(45.59%)	9772507(22.52%)	29699851(68.45%)	37903320(87.36%)
YAN25_1	40312540	36663686(90.95%)	36412837(90.33%)	250849(0.62%)	18197001(45.14%)	18215836(45.19%)	18156177(45.04%)	18256660(45.29%)	8305684(20.6%)	28107153(69.72%)	34887874(86.54%)
YAN25_2	51163252	46458408(90.8%)	46121868(90.15%)	336540(0.66%)	23110818(45.17%)	23011050(44.98%)	22992708(44.94%)	23129160(45.21%)	10299592(20.13%)	35822276(70.02%)	43900912(85.81%)
YAN25_3	39508866	35866245(90.78%)	35611963(90.14%)	254282(0.64%)	17828487(45.13%)	17783476(45.01%)	17754354(44.94%)	17857609(45.2%)	8330195(21.08%)	27281768(69.05%)	34153622(86.45%)
PB25_1	45605730	41294451(90.55%)	40974377(89.84%)	320074(0.7%)	20550647(45.06%)	20423730(44.78%)	20408144(44.75%)	20566233(45.1%)	9852696(21.6%)	31121681(68.24%)	39069468(85.67%)
PB25_2	44507278	40078764(90.05%)	39775368(89.37%)	303396(0.68%)	19942087(44.81%)	19833281(44.56%)	19802447(44.49%)	19972921(44.88%)	9632438(21.64%)	30142930(67.73%)	37908766(85.17%)
PB25_3	44563098	39754369(89.21%)	39362917(88.33%)	391452(0.88%)	19743263(44.3%)	19619654(44.03%)	19620662(44.03%)	19742255(44.3%)	9345783(20.97%)	30017134(67.36%)	37345662(83.8%)
